# White matter hyperintensities and TDP‐43 pathology in Alzheimer's disease

**DOI:** 10.1002/alz.14516

**Published:** 2025-01-17

**Authors:** Arenn F. Carlos, Stephen D. Weigand, Nha Trang Thu Pham, Ronald C. Petersen, Clifford R. Jack, Dennis W. Dickson, Jennifer L. Whitwell, Keith A. Josephs

**Affiliations:** ^1^ Department of Neurology Mayo Clinic Rochester Minnesota USA; ^2^ Department of Quantitative Health Sciences Mayo Clinic Rochester Minnesota USA; ^3^ Department of Radiology Mayo Clinic Rochester Minnesota USA; ^4^ Department of Neuroscience (Neuropathology) Mayo Clinic Jacksonville Florida USA

**Keywords:** Alzheimer's disease, FLAIR, FTLD, LATE_NC, TDP‐43

## Abstract

**INTRODUCTION:**

Greater white matter hyperintensities (WMHs) on magnetic resonance imaging (MRI) are seen with transactive response DNA‐binding protein 43 (TDP‐43) pathology in frontotemporal lobar degeneration (FTLD‐TDP). WMH associations with TDP‐43 pathology in Alzheimer's disease (AD‐TDP) remain unclear.

**METHODS:**

A total of 157 participants from Mayo Clinic Rochester with autopsy‐confirmed AD, known TDP‐43 status, and antemortem fluid‐attenuated inversion recovery (FLAIR) MRI were included. Vascular risk factors were assessed. A semi‐automated WMH segmentation‐quantification process produced total and regional WMH volumes. Penalized linear regression models adjusting for age at MRI analyzed TDP‐43 associations (status and typing) with WMHs.

**RESULTS:**

TDP‐43‐positive status was not associated with WMH burden overall because opposite effects were seen based on AD‐TDP typing. Despite similar antemortem vascular risk factors and postmortem vascular pathologies, AD‐TDP type‐*α* showed greater total and regional WMH burden (particularly in subcortical frontotemporal and basal ganglia regions) than TDP‐43 negatives and AD‐TDP type‐*β*.

**DISCUSSION:**

AD‐TDP types may have different WMH pathomechanisms, with type‐*α* having associations more like FTLD‐TDP than AD.

**Highlights:**

- In transactive response DNA‐binding protein 43 (TDP‐43) pathology in Alzheimer's disease (AD‐TDP), TDP‐43 status alone is not associated with total or regional WMH burden
- AD‐TDP type‐*α* shows greater total, frontotemporal subcortical, and basal ganglia white matter hyperintensities (WMHs)
- AD‐TDP type‐*β* shows less total and subcortical occipital WMHs
- AD‐TDP type‐*α* effect on WMH burden closely mimics the effects of frontotemporal lobar degeneration with TDP‐43 (FTLD‐TDP) rather than AD
- Different relationships of AD‐TDP types with WMHs suggest different pathomechanisms

## 1 BACKGROUND

The term “leukoaraiosis” was coined in the late 1980s to describe a phenomenon of white matter rarefaction of the aging brain that presented on magnetic resonance imaging (MRI) as white matter hyperintensities (WMHs) or on computed tomography (CT) as lucencies.^1^, ^2^ These WMHs were typically found in the periventricular and subcortical regions and were believed to derive from cerebrospinal fluid (CSF)–filled dilated perivascular spaces surrounding normal small arteries and veins resulting from cortical atrophy and axonal rarefaction.^2^, ^3^, ^4^ Other neuropathological correlates of WMHs included predominantly small vessel disease, particularly arteriosclerosis and lacunar infarcts, as well as myelin and axonal loss, gliosis, and spongiosis, whereas the etiology included ischemia, hypoperfusion, inflammation, and impaired blood–brain barrier (BBB) integrity.^5^, ^6^, ^7^, ^8^, ^9^, ^10^ More recent studies have found associations between WMHs and neurodegenerative diseases, including Alzheimer's disease (AD), where the bulk of WMHs is located in the parieto‐occipital regions,^10^, ^11^, ^12^, ^13^, ^14^, ^15^ and frontotemporal lobar degeneration (FTLD), where WMHs are most frequent in the frontotemporal distribution.^12^, ^16^, ^17^, ^18^

Transactive response DNA‐binding protein 43 (TDP‐43) forms pathological inclusions in normal aging^19^, ^20^ and in neurodegenerative diseases, including AD^21^, ^22^ and FTLD.^23^, ^24^ In AD with TDP‐43 pathology (AD‐TDP), there are two main types of TDP‐43 inclusions: type‐*α*, where inclusions mimic those seen in FTLD with TDP‐43 (FTLD‐TDP) and include neuronal cytoplasmic inclusions (NCIs), neuronal intranuclear inclusions (NIIs), dystrophic neurites (DNs), fine neurites of the hippocampus (FNs), and perivascular inclusions (PVs); and type‐*β*, where TDP‐43 co‐deposits with tau neurofibrillary tangles (NFTs) in the setting of elevated AD neuropathologic changes (ADNCs).^25^, ^26^ Relationships between TDP‐43 pathology and WMHs have insofar been studied mostly in the context of FTLD and less in AD. In FTLD‐TDP, not only are significant white matter changes (gliosis and spongiosis) seen at postmortem evaluation, but significant frontotemporal WMH burden is also observed on neuroimaging independent of old age.^18^, ^27^, ^28^, ^29^ Although AD‐TDP more frequently occurs with increasing age^30^, ^31^, ^32^ and has also been strongly associated with moderate to severe arteriolosclerosis,^33^, ^34^ little is known about the severity of WMHs or other neuroimaging markers of white matter damage in AD‐TDP. A recent study assessed white matter health through neurite density index and free water measures in AD‐TDP participants identified through antemortem fluorodeoxyglucose–positron emission tomography (FDG‐PET)–based TDP‐43 signature and found correlations between temporal white matter degeneration and TDP‐43 pathology.^35^ Another study using diffusion tensor imaging (DTI) showed that lower diffusion anisotropy in medial temporal lobe was associated with more widespread AD‐TDP burden.^36^ Although several studies have assessed associations between WMH burden and AD with or without TDP‐43 pathology, these studies have focused mainly on the relationship between WMH burden and AD pathological hallmarks, including amyloid‐β (Aβ) and NFTs.^11^, ^14^, ^15^ To our knowledge, no study has yet analyzed the potential associations between WMH burden and TDP‐43 pathology in AD‐TDP.

In this study, our primary aim was to determine the possible associations between total and regional WMH burden and TDP‐43 status. Given that AD‐TDP type‐*α* more closely resembles FTLD‐TDP and type‐*β* is tightly associated with higher ADNCs, our secondary aim was to investigate whether WMH signature would also vary by AD‐TDP typing. We hypothesized that, like FTLD‐TDP, greater total and regional WMH burden in the frontal and temporal lobes would be found and would be associated with AD‐TDP type‐*α*.

## 2 METHODS

### 2.1 Design, setting, and participants

This cross‐sectional, clinico‐radio‐pathologic study was performed at the Mayo Clinic in Rochester, MN, USA. Participants were prospectively recruited and followed in one of three National Institutes of Health (NIH)–funded entities: the Mayo Clinic Alzheimer's Disease Research Center, the Mayo Clinic Study of Aging, and the Neurodegenerative Research Group. Participants had died between November 2006 and July 2021. All participants underwent standardized neurologic, neuroimaging, and neuropathologic evaluations. To be included in the study, participants must have had: (1) an antemortem brain T2‐weighted and two‐dimensional (2D) fluid‐attenuated inversion recovery (FLAIR) MRI scan obtained within 5 years of death and using a GE scanner; and (2) a postmortem neuropathologic evaluation to establish ADNC and TDP‐43 pathology status. Participants with a pathological diagnosis of FTLD, with either TDP‐43, tau, or FET (Fused in sarcoma, Ewing sarcoma, and TATA‐binding protein‐associated factor 2N) proteins were excluded. A total of 157 participants were included in this study.

### 2.2 Clinical evaluation

All participants underwent standardized neurological and neuropsychological evaluation, as described previously.^37^, ^38^ A diagnosis of cognitive state (cognitively unimpaired, mild cognitive impairment, or dementia) was determined following a consensus meeting and was based on all available clinical/neuropsychological data.

Electronic medical records of participants were also reviewed for vascular risk factors that could affect WMH burden. These included the presence of hypertension, hyperlipidemia, diabetes mellitus, and tobacco use.

### 2.3 MRI image acquisition

All MRI was obtained on a 3.0 T GE scanner (GE Healthcare), using a protocol that included a T1‐weighted three‐dimensional (3D) magnetization‐prepared rapid acquisition gradient echo (MPRAGE) performed using the following parameters: repetition time (TR) = 2300 ms, echo time (TE) = 3 ms, inversion time (TI) = 900 ms; 8° flip angle, 26‐cm field of view; 256 × 256 in‐plane matrix with a phase field of view of 0.94, and 1.2 mm slice thickness; and 2D T2‐FLAIR performed using the following parameters: TR = 11,000 ms, TE = 147 ms, TI = 2250 ms, 24 cm field of view, 256 × 192 in‐plane matrix, and 3 mm slice thickness.

### 2.4 MRI image processing and analysis

Both MPRAGE and FLAIR images were used for a semi‐automated WMH segmentation and quantification of WMH volumes. All FLAIR images initially underwent an automated segmentation process using an in‐house software, as described previously.^11^ In this process, possible WMH voxels were first identified through clustering via connected components using the FLAIR images. The FLAIR images were then aligned to statistical parametric mapping (SPM) 12 segmentations from T1‐weighted images to obtain brain masks that allowed identification and removal of nonbrain tissue or voxels that were more likely to be gray matter and less likely to be WMHs. The resulting WMH masks were then manually inspected and edited by a trained imaging analyst to further ensure correct and consistent WMH identification. The WMH masks were also inspected for possible infarctions which, when identified, were manually edited out. Separate regional WMH volumes were obtained using an in‐house 22‐region atlas, which identified left and right subcortical and periventricular WMH within the main cerebral lobes (frontal, temporal, parietal, and occipital), as well as WMHs within the left and right corpus callosum, deep gray and white matter, brainstem, and cerebellum, as described previously.^13^ For this study, our focus was on nine specific regions of interest that are among those most affected by AD‐TDP.^39^, ^40^ This included four periventricular regions from the frontal, temporal, parietal, and occipital lobes; four subcortical regions from the frontal, temporal, parietal, and occipital lobes; and the basal ganglia. This last region consists of both deep gray and white matter subregions, and studies have shown that WMH and impaired white matter connectivity in these areas were associated with not only vascular dementia but also neurodegenerative diseases, such as AD and TDP‐43 pathology^36^, ^41^, ^42^, ^43^; hence, the basal ganglia was included in the study. Total intracranial volume (TIV) was obtained for all participants.

**RESEARCH IN CONTEXT**

1. **Systematic review**: The relationship between white matter hyperintensity (WMH) burden and transactive response DNA‐binding protein 43 (TDP‐43) pathology in frontotemporal lobar degeneration (FTLD‐TDP) has been studied extensively, yet associations with TDP‐43 pathology in Alzheimer's disease (AD‐TDP) remain unclear. We reviewed the literature using PubMed for studies assessing WMH burden and other measures of white matter damage in AD‐TDP.
2. **Interpretation**: Our findings provide evidence that TDP‐43 pathology in AD‐TDP correlates with WMH burden; yet opposite effects are seen based on AD‐TDP typing (type‐*α* and type‐*β*), which leads us to hypothesize whether the two main types of AD‐TDP could represent different pathomechanisms, particularly with type‐*α* closely mimicking the effects of FTLD‐TDP rather than AD.
3. **Future directions**: Future studies should (1) validate our results and investigate the potential use of WMH quantification/scoring as an adjunct antemortem neuroimaging biomarker for AD‐TDP, and (2) further investigate the different pathomechanisms and relationships with white matter damage associated with various AD‐TDP types.

The WMH volumes (measured in mm^3^) from the left and right hemispheres were summed to obtain a “regional” WMH volume from each region of interest from the periventricular and subcortical frontal, temporal, parietal, and occipital lobes and the deep gray and white matter of the basal ganglia. The resulting regional WMH volumes from all the abovementioned regions were then summed to yield a “total” WMH volume. All WMH volumes (both reginal and total) were divided by TIV to correct for differences in head sizes and obtain a WMH percentage (WMH%).

### 2.5 Neuropathological analysis

Postmortem neuropathologic examination was conducted on all participants. The amygdala is the first region affected by AD‐TDP^39^, ^40^ and therefore, all were initially screened for TDP‐43 pathology using amygdala sections immunostained with a monoclonal anti‐phosphorylated TDP‐43 antibody (pS409/420, 1:500, Cosmo Bio, Tokyo, Japan). None of the participants showed changes suggestive of FTLD, including evidence of focal frontal and/or temporal neurodegeneration, whether seen macroscopically with gross atrophy or microscopically with neuronal loss, microvacuolation, and gliosis.^44^ Cases showing TDP‐43 immunoreactive inclusions within the parenchyma of the amygdala were considered TDP‐43‐positive (TDP‐43(+)) and were further subjected to TDP‐43 typing. TDP‐43(+) cases showing FTLD‐like inclusions, including NCIs, NIIs, or DNs were classified as “type‐*α*”; cases showing “apple‐bite” or “flame‐shaped” tangle‐associated TDP‐43 or TATs (consisting of TDP‐43 colocalizing with paired helical tau filaments)^26^ were classified as “type‐*β*.”^25^ The TDP‐43(+) were subjected to TDP‐43 staging using the Josephs 6‐stage scheme^39^, ^40^ by analyzing additional sections from the medial temporal lobe, basal forebrain, brainstem regions, basal ganglia, and neocortex. A composite TDP‐43 staging was used whereby cases with TDP‐43 stage 1 were classified as “amygdala‐only”, stages 2–3 as “limbic,” and stages 4–6 as “extra‐limbic.”

The severity of ADNCs was determined following the recommendations of the National Institute on Aging‐Reagan Criteria^45^ or the National Institute on Aging–Alzheimer's Association (NIA‐AA)^46^ guidelines. To determine AD pathology, pertinent brain sections were stained with modified Bielschowsky silver stain to assess for NFTs and neuritic plaques and with an anti‐Aβ antibody (clone 6F/3D, 1:10; Novocastra Vector Labs, Burlingame, CA) to assess for amyloid plaques. Cerebrovascular burden was established using sections stained with hematoxylin and eosin (H&E) to assess for the presence and severity of cerebral amyloid angiopathy (CAA) (supplemented with Aβ histochemistry), arteriolosclerosis, microinfarcts, and lacunar (<1 cm)/large infarcts (>1 cm). The severity of CAA and arteriolosclerosis was initially graded on a scale of 0–3, for absent, mild, moderate and severe. A five‐point vascular composite score was consequently obtained, as described previously.^47^

### 2.6 Statistical methods

Categorical variables were described using counts and percentages and were analyzed using Chi‐square tests or Fisher's exact tests, as appropriate. Continuous variables were described as medians and first and third quartiles (Q1,Q3) and were analyzed using Wilcoxon rank‐sum tests.

We used linear regression methods to estimate associations between AD‐TDP and WMH volume. The dependent variable for these models was log‐transformed WMH volume as a percentage of TIV. Models were adjusted for age at MRI. For one set of analyses, we estimated the effect of TDP‐43(+) status based on total WMH burden or regional WMH burden, fitting separate models for each region. For another set of analyses, we included only the subset of individuals who had AD‐TDP type‐*α* lesions, AD‐TDP type‐*β* lesions, or were negative for TDP‐43 pathology (TDP‐43(‐)), and we compared WMH burden for type‐*α* versus TDP‐43(−), type‐*β* versus TDP‐43(−), and type‐*α* versus type‐*β*.

Because we performed separate analyses of total WMH burden and regional WMH, we used penalized maximum likelihood to stabilize our estimates and reduce false‐positive findings.^48^, ^49^ Our penalization was equivalent to specifying a 95% prior probability that regression coefficients corresponding to TDP‐43 differences (i.e., effect sizes) were within ± 1. This approach reduces bias by shrinking estimates toward the null value and improves generalizability.^50^, ^51^ Because models included a log‐transformed response, regression estimates were exponentiated so that effects could be interpreted in terms of a ratio of geometric means, or relative WMH burden.^52^ All statistical analyses were performed using R version 4.4.1 with penalized maximum likelihood performed using the **bayesglm** function in the **arm** package. *p*‐values < 0.05 were considered statistically significant.

## 3 RESULTS

### 3.1 Participant characteristics

Of the 157 total participants, 78 (50%) were TDP‐43(+). The demographic, clinical, and neuropathological features of the cohort stratified by TDP‐43 status are shown in Table 1. There was only a trend for the TDP‐43(+) to be older at MRI and at death (*p *= 0.08 and *p *= 0.09, respectively). Clinically, TDP‐43(+) cases were more likely to have dementia at the time of death than their TDP‐43(−) counterparts (69% vs 51%, *p *= 0.01). There were no statistically significant differences in the frequency of antemortem vascular risk factors or total WMH burden between the two groups. From a pathological standpoint, AD‐TDP pathology was limited to the amygdala in 43%, involved the limbic system (entorhinal and hippocampal regions) in 25%, and extended into extra‐limbic regions (basal ganglia, brainstem, frontal neocortex) in 32% of the TDP‐43(+) cases. Finally, the TDP‐43(+) cases more frequently had intermediate to high ADNCs (*p *= 0.001), but there was no difference in the vascular composite score (or its individual components; see Table S1)

**TABLE 1:** Demographic, clinical, and neuropathological characteristics of study participants stratified by TDP‐43 status.

Characteristics	TDP‐43(−) (*n* = 79)	TDP‐43(+) (*n* = 78)	*p*‐value
**Female**	26/79 (33%)	33/78 (43%)	0.22
**Education, years**	14 (12, 18)	16 (12, 18)	0.17
**Age at MRI, years**	79.0 (71.2, 87.1)	82.7 (74.6, 88.8)	0.09
**Age at death, years**	82.1 (73.6, 90.1)	85.6 (77.1, 90.9)	0.08
**Time from scan to death, years**	2.4 (1.3, 3.2)	2.6 (1.2, 4.0)	0.23
**Cognitive status**			**0.01**
Normal	25/69 (36%)	10/71 (14%)	
MCI	9/69 (13%)	12/71 (17%)	
Dementia	35/69 (51%)	49/71 (69%)	
**Vascular risk factors**			
Hypertension	49/59 (83%)	41/56 (73%)	0.20
Hyperlipidemia	46/59 (78%)	38/56 (68%)	0.22
Diabetes mellitus	18/59 (31%)	11/56 (20%)	0.20
Tobacco use	13/45 (29%)	23/50 (46%)	0.10
**TIV, cm^3^**	1551.6 (1422.7, 1646.8)	1532.4 (1383.5, 1653.0)	0.28
**Total WMH, mm3**	20247.1 (12,286.1, 40454.3)	23884.2 (13492.5, 40223.0)	0.52
**Total WMH, %**	1.3 (0.8, 2.4)	1.5 (0.9, 2.8)	0.42
**TDP‐43 composite stage**			
Amygdala‐only		24/56 (43%)	
Limbic		14/56 (25%)	
Extra‐limbic		18/56 (32%)	
**ADNC**			**0.001**
Not	6/47 (13%)	4/43 (9%)	
Low	16/47 (34%)	2/43 (5%)	
Intermediate	7/47 (15%)	18/43 (42%)	
High	18/47 (38%)	19/43 (44%)	
**Vascular composite score**			0.64
0	0/68 (0%)	1/71 (1%)	
1	7/68 (10%)	4/71 (6%)	
2	33/68 (49%)	36/71 (51%)	
3	8/68 (12%)	6/71 (8%)	
4	20/68 (29%)	24/71 (34%)	

*Note*: Data are shown as counts (%) or median (Q1, Q3). *p*‐values are from the chi‐square test, Fisher's exact test, or Wilcoxon rank‐sum, as appropriate. Total WMH volume is given by the sum of regional WMH volumes in the left and right frontal, temporal, parietal, and occipital lobes and left and right basal ganglia WMH % is given by the ratio between WMH volume and TIV. The *p*‐values in bold are significant.

Abbreviations: ADNCs, Alzheimer's disease neuropathologic changes; MCI, mild cognitive impairment; MRI, magnetic resonance imaging; TDP‐43, transactive response DNA‐binding protein 43; TIV, total intracranial volume; WMH, white matter hyperintensity.

Of the TDP‐43(+) participants, 63% showed type‐*α* inclusions and the remaining 37% showed type‐*β*. The characteristics of TDP‐43(+) participants stratified by AD‐TDP typing are shown in Table 2. There were no statistically significant differences in demographic characteristics except for a trend for type‐*β* to have longer years of education (*p *= 0.07). The two groups were similar in terms of cognitive status. Although there were no differences in antemortem vascular risk factors or postmortem vascular composite scores (or its components; see Table S1), type‐*α* had almost double the amount of WMH burden compared to type‐*β* (29.0 cm^3^ vs 16.2 cm^3^, *p *= 0.02). Pathologically, there was also a trend for type‐*α* to show more widespread TDP‐43 pathology (*p *= 0.09).

**TABLE 2:** Demographic, clinical, and neuropathological characteristics of study participants stratified by TDP‐43 typing.

Characteristics	Type‐*α* (*n *= 25)	Type‐*β* (*n* = 15)	*p‐*value
**Female**	11/25 (44%)	5/15 (33%)	0.51
**Education, years**	14 (12, 16)	17 (15, 19)	0.07
**Age at MRI, years**	82.7 (76.7, 88.0)	84.6 (76.9, 88.9)	0.76
**Age at death, years**	85.6 (79.6, 89.8)	86.9 (78.1, 91.2)	0.78
**Time from scan to death, years**	2.0 (0.9, 3.8)	1.5 (1.1, 3.4)	0.98
**Cognitive status**			0.64
Normal	2/24 (8%)	2/14 (14%)	
MCI	3/24 (13%)	3/14 (21%)	
Dementia	19/24 (79%)	9/14 (64%)	
**Vascular risk factors**			
Hypertension	13/15 (87%)	9/12 (75%)	0.63
Hyperlipidemia	9/15 (60%)	9/12 (75%)	0.68
Diabetes mellitus	2/15 (13%)	2/12 (17%)	>0.99
Tobacco use	6/11 (55%)	2/10 (20%)	0.18
**TIV, cm^3^**	1521.3 (1404.4, 1635.8)	1589.9 (1403.6, 1619.6)	0.78
**Total WMH, mm^3^**	29045.7 (19800.6, 54281.4)	16184.8 (9979.5, 30241.7)	**0.02**
**TDP‐43 composite stage**			0.09
Amygdala‐only	6/23 (26%)	6/10 (60%)	
Limbic	3/23 (13%)	2/23 (20%)	
Extra‐limbic	14/23 (61%)	2/10 (20%)	
**ADNC**			0.30
Not	3/14 (21%)	0/7 (0%)	
Low	1/14 (7%)	1/7 (14%)	
Intermediate	5/14 (36%)	5/7 (71%)	
High	5/15 (36%)	1/7 (14%)	
**Vascular composite score**			>0.99
0	1/25 (4%)	0/14 (0%)	
1	1/25 (4%)	1/14 (7%)	
2	12/25 (48%)	7/14 (50%)	
3	2/25 (8%)	1/14 (7%)	
4	9/25 (36%)	5/14 (36%)	

*Note*: Data are shown as counts (%) or median (Q1, Q3). *p*‐values are from the chi‐square test, Fisher's exact test, or Wilcoxon rank‐sum, as appropriate. Total WMH volume is given by the sum of regional WMH volumes in the left and right frontal, temporal, parietal, and occipital lobes and left and right basal ganglia.

Abbreviation: ADNCs, Alzheimer's disease neuropathologic changes; MCI, mild cognitive impairment;TDP‐43, transactive response DNA‐binding protein 43; TIV, total intracranial volume; WMH, white matter hyperintensity.

### 3.2 WMH burden and TDP‐43 inclusions

**FIGURE 1:**
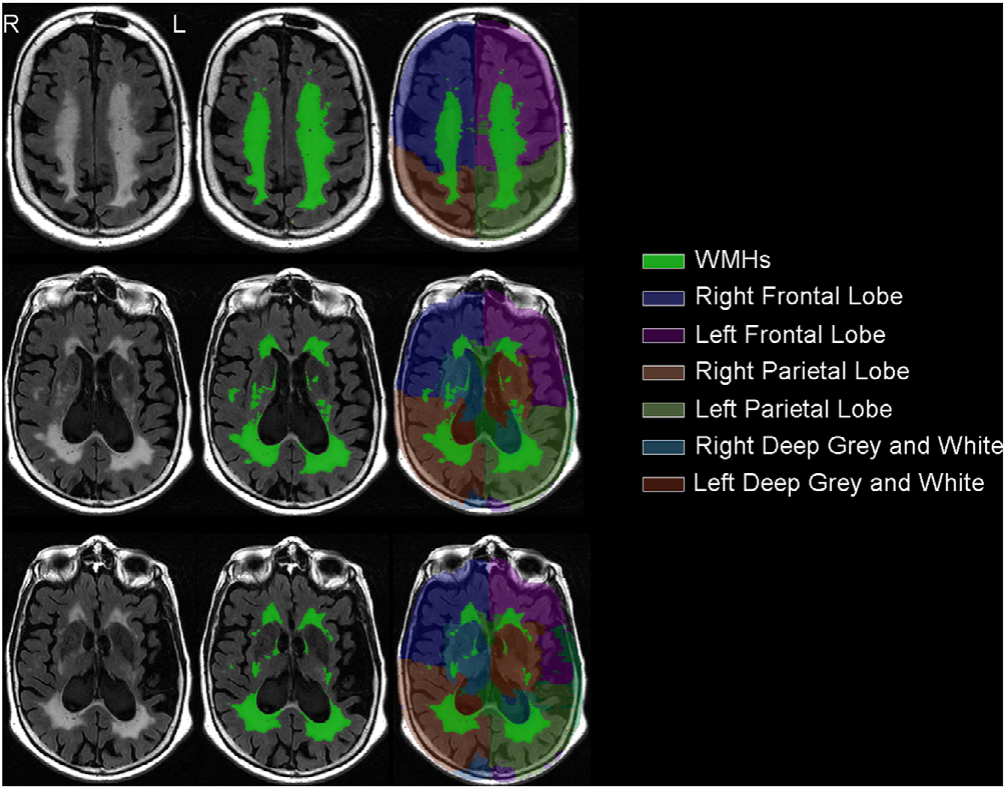
White matter hyperintensity identification and segmentation. Representative images showing 2D FLAIR MRI with superimposed WMH mask and WMH segmentation are shown. WMHs are identified in green, whereas the segmentation of the cortical lobes and deep gray and white matter of the basal ganglia are shown in the accompanying color atlas. 2D FLAIR, two‐dimensional fluid‐attenuated inversion recovery; MRI, magnetic resonance imaging; WMH, white matter hyperintensity.

**FIGURE 2 alz14516-fig-0002:**
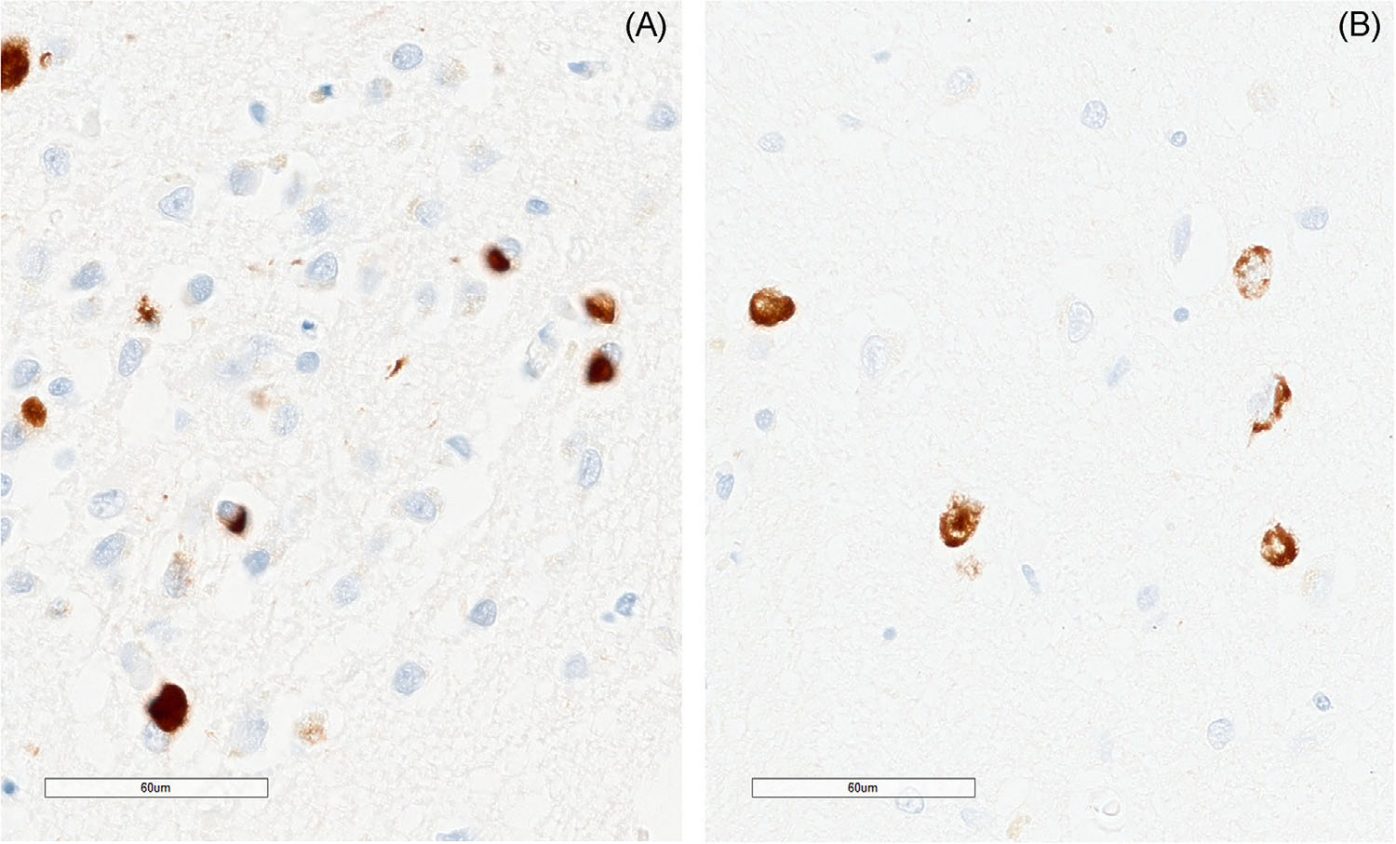
TDP‐43 pathology types in AD. Representative images show the two major types of AD‐TDP pathology: type‐*α*, with NCIs and DNs (A) and type‐*β* with TATs (B). Scale bar = 60 µm. AD‐TDP, Alzheimer's disease with TDP‐43 pathology; DN, dystrophic neurite; NCI, neuronal cytoplasmic inclusion; TAT, tangle‐associated TDP‐43.

**FIGURE 3 alz14516-fig-0003:**
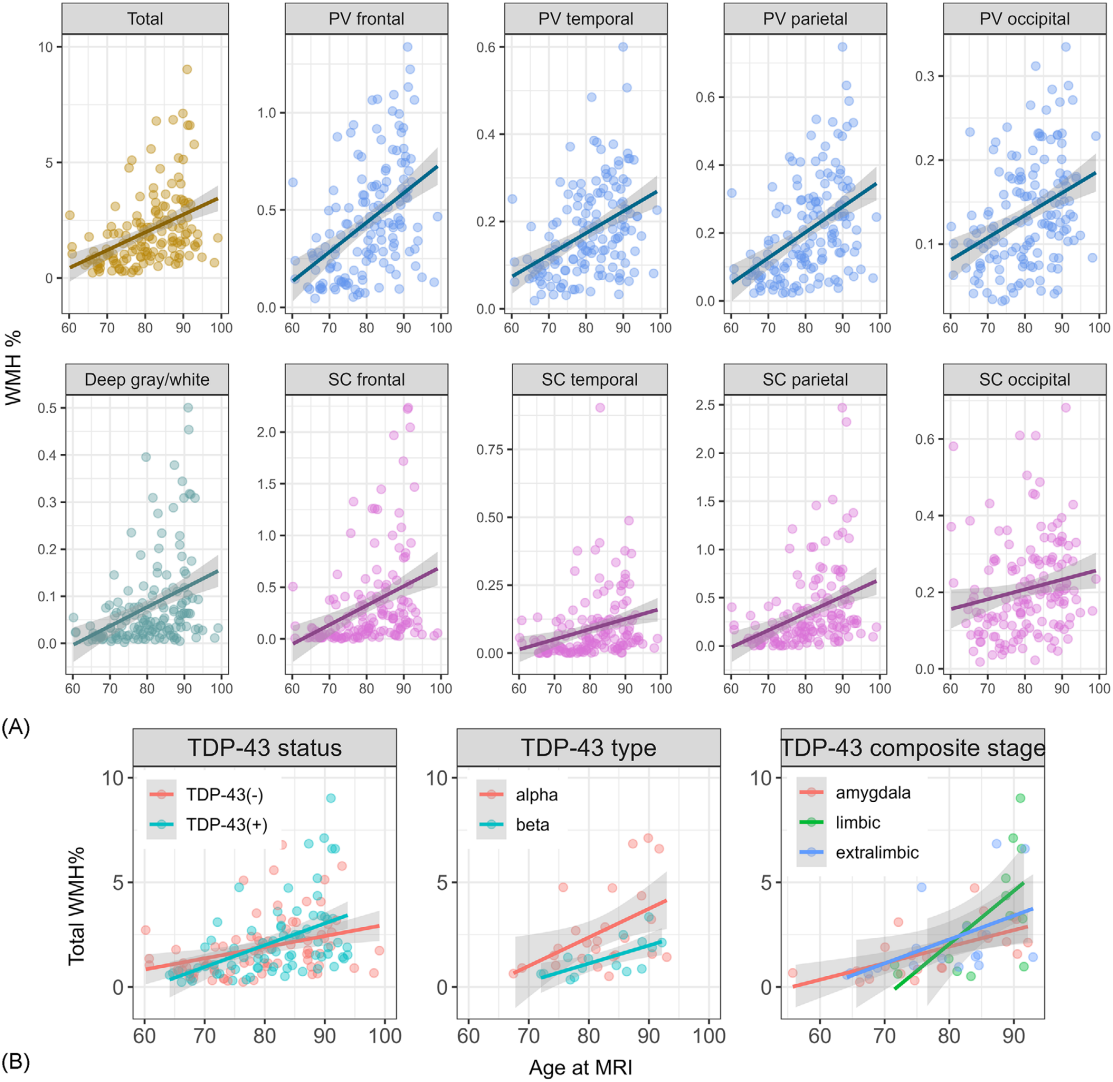
Distribution of total and regional WMH data and relationship with TDP‐43 pathology. Total and regional WMH% (given by WMH volumes/total intracranial volume) are shown in (A). The regional WMH% are from the periventricular and subcortical frontal, temporal, parietal, and occipital lobes, and the basal ganglia. The distribution of total WMH% separated by TDP‐43 status, typing, and staging is shown in (B). PV, periventricular; SC, subcortical; TDP‐43, transactive response DNA‐binding protein 43; WMH, white matter hyperintensity.

Figure 1 shows representative images of WMH with the corresponding masks and semi‐automated segmentation. The two main types of TDP‐43 inclusions, type‐*α* and type‐*β*, are shown in Figure 2A,B. Scatter plots in Figure 3 show the distribution of data representing total and regional WMH% in all cohorts. The distribution of total WMH% stratified by TDP‐43 status, TDP‐43 type, and TDP‐43 composite staging is also shown. WMH burden in all regions increased with age.

### 3.3 TDP‐43 status effect on total and regional WMH burden

**FIGURE 4 alz14516-fig-0004:**
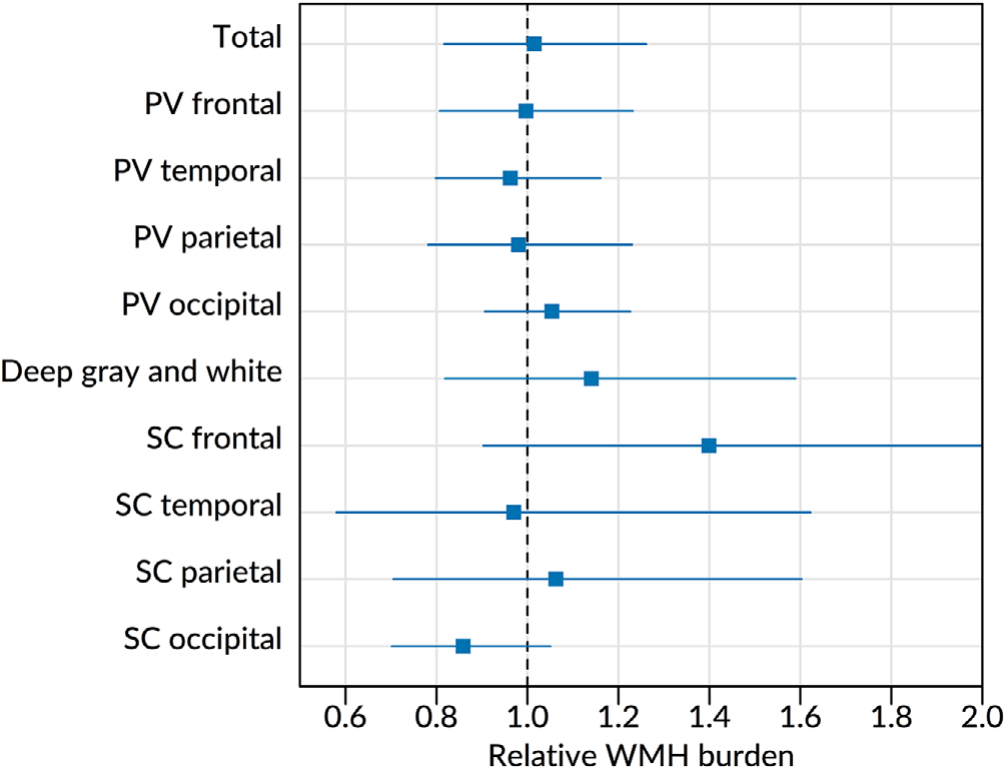
WMH burden and TDP‐43 status. The forest plots show the estimated relative WMH burden (relative geometric means) for total and regional WMH volumes associated with TDP‐43(+) versus TDP‐43(‐) status. The dots represent the estimated effects, whereas the lines represent 95% CIs. Upper CIs not crossing the line of null effect (vertical line at /*x*/ = 1) are considered significant at *p* < 0.05. Some upper confidence limit clipped (see Table S2). CI, confidence interval; PV, periventricular; SC, subcortical; TDP‐43, transactive response DNA‐binding protein 43; WMH, white matter hyperintensity.

There were no significant differences in total or regional WMH burden between TDP‐43(+) and TDP‐43(−) participants (Figure 4 and Table S2).

### 3.4 TDP‐43 type effect on total and regional WMH burden

#### 3.4.1 Type‐*α* relative to TDP‐43 negative

**FIGURE 5 alz14516-fig-0005:**
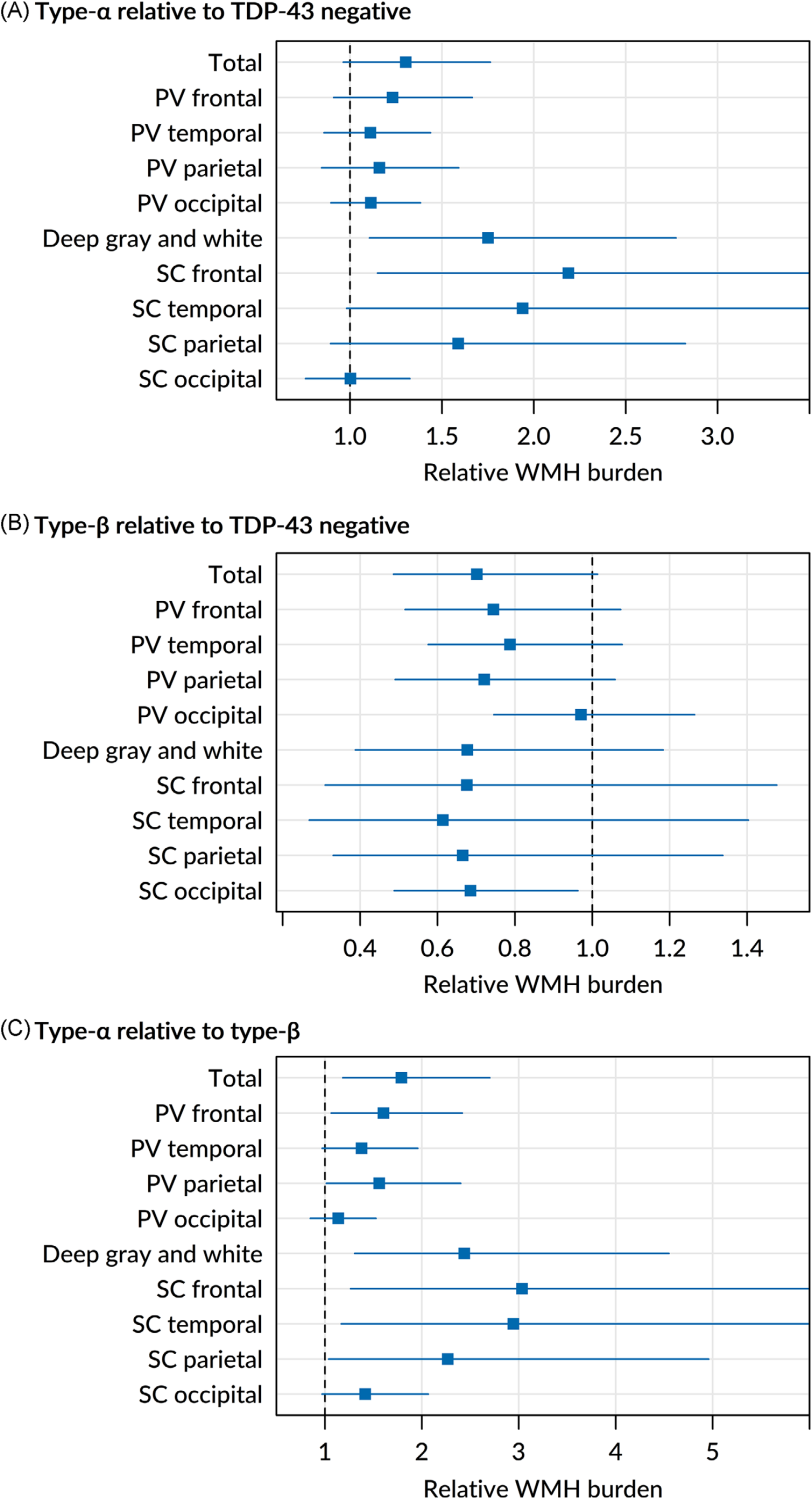
WMH burden and TDP‐43 type. The forest plots show the estimated relative WMH burden (relative geometric mean) for total and regional WMH volumes associated with different types of AD‐TDP inclusions. The effects of type‐*α* relative to TDP‐43(−) are shown in A, those of type‐*β* relative to TDP‐43(−) are shown in B, and those of type‐*α* relative to type‐*β* are shown in C. The dots represent the estimated effects, whereas the lines represent 95% CIs. Some upper confidence limit clipped (see Table S3). CIs not crossing the line of null effect (vertical line at /*x*/ = 1) are considered statistically significant at *p* < 0.05. CI, confidence interval; PV, periventricular; SC, subcortical; TDP‐43, transactive response DNA‐binding protein 43; WMH, white matter hyperintensity.

Type‐*α* showed an overall trend for greater total WMH burden (+30%, *p *= 0.09) compared to TDP‐43(−) participants (Figure 5A). This was driven by the significantly greater WMH volumes in the subcortical frontal region (greater than 2‐fold relative burden, *p *= 0.02) and basal ganglia (increased by 75%, *p *= 0.02). There was also some evidence for greater subcortical temporal WMH burden (increased by 94%, *p *= 0.06).

#### 3.4.2 Type‐*β* relative to TDP‐43 negative

An overall pattern of less WMH burden was seen in participants with type‐*β* compared to TDP‐43(–) (Figure 5B). Specifically, a trend for about 30% less total WMH burden was seen in type‐*β* participants (*p *= 0.06). However, although most regions (except for the periventricular occipital region) generally displayed less regional WMH burden, only the subcortical occipital region showed a significant change (−30%, *p *= 0.03) with a trend seen for the periventricular parietal region (−30%, *p *= 0.09).

#### 3.4.3 Type‐*α* relative to type‐*β*

Participants with type‐*α* pathology showed about 80% greater total WMH burden (*p *= 0.01) compared to type‐*β*, with widespread contributions from regional WMH volumes in the periventricular, subcortical, and basal ganglia regions (Figure 5C). About 60% bigger WMH volumes were found in the periventricular frontal and parietal lobes (*p *= 0.02 and *p *= 0.04, respectively), with a trend for about 40% increase in the periventricular temporal lobe (*p *= 0.07). The deep gray and white matter regions of the basal ganglia also showed more than twice the WMH burden in type‐*α* compared to type‐*β* (*p *= 0.01). The subcortical regions displayed the most substantial increase[Fig alz14516-fig-0002] in WMH burden, with about a 3‐fold increase in the frontal and temporal lobes (*p *= 0.01 and *p *= 0.02, respectively) and more than a 2‐fold increase in the parietal lobe (*p *= 0.04). The occipital lobe appears to be less involved, with the periventricular occipital lobe being the only region that did not show any increase or decrease in WMH burden and with the subcortical occipital lobe showing only a trend for about 40% increase in WMH volumes (*p *= 0.07). Detailed results from the analyses involving AD‐TDP typing are shown in Table S3.

## 4 DISCUSSION

In this study, we found that the presence alone[Fig alz14516-fig-0003] of concomitant[Fig alz14516-fig-0004] TDP‐43 pathology in AD was not associated with decreases or increases in WMH burden, as two opposite effects were observed based on the type of pathologic inclusion. Our most important finding is the strong relationship between AD‐TDP type‐*α* and increased WMH burden. Indeed, not only did participants with type‐*α* inclusions show evidence of greater total and regional WMH burden (particularly in the subcortical frontotemporal lobe and basal ganglia) compared to TDP‐43(−), but they further showed considerably and extensively greater WMH burden than TDP‐43(+) participants with type‐*β* inclusions. These findings would suggest different pathomechanisms involving the two distinct AD‐TDP types.

The overall characteristics of AD‐TDP type‐*α* are reminiscent of those seen in FTLD‐TDP type A,^25^ where NCIs, NIIs, short DNs, FNs, PVs, and oligodendroglial inclusions are seen in frontotemporal cortical regions and subcortical regions such as the basal ganglia, amygdala, and thalamus.^26^, ^53^, ^54^, ^55^, ^56^ Due to similarities in inclusion characteristics, it is not surprising that our findings align with several previous studies on WMH burden and FTLD‐TDP.^12^, ^16^, ^18^, ^27^, ^57^ These studies indeed found a relationship between greater WMH burden and pathologically confirmed FTLD‐TDP type A^16^, ^18^, ^27^ or frontotemporal dementia (FTD) cases with progranulin (*GRN*) mutations,^27^, ^41^, ^57^, ^58^, ^59^ which typically present with underlying FTLD‐TDP type A pathology.^53^, ^54^ Some studies, however, also found associations with FTD cases with *c9orf72* mutations,^41^, ^57^, ^60^ which typically correlate with FTLD‐TDP type B pathology.^53^, ^54^ Like our findings, these studies found the highest WMH burden in the frontotemporal lobes, coinciding with the regions with the most atrophy.^16^, ^18^, ^41^, ^57^ Although we did not measure MRI gray and white matter volumes (as this is beyond the scope of the current study), previous structural MRI studies have demonstrated atrophy in the medial temporal and frontal lobes of patients with AD‐TDP.^31^, ^40^, ^61^ Although the studies on FTLD‐TDP did not show involvement of the parietal lobes, they did reveal the predominance of subcortical rather than periventricular WMHs as our findings.^18^, ^27^, ^41^ Comparing our study with existing white matter damage studies in AD‐TDP is challenging due to different study designs. Nonetheless, our findings have similarities with those of Tazwar et al.,^36^ who used transverse relaxation time (R2) (which describes the rate of decay of transverse magnetization) to detect disruptions in temporo‐parietal, fronto‐temporal, and temporo‐basal ganglia white matter connectivity associated with gray matter TDP‐43 burden, granting that this study only investigated overall TDP‐43 status. Finally, another study by Raghavan et al.^35^ revealed temporal white matter damage using neurite orientation dispersion and density imaging measures in patients with suspected TDP‐43 pathology based on FDG‐PET‐based TDP‐43 signature.

It is well known that the severity of WMH is closely related to the presence of vascular risk factors, including old age, female sex, hypertension, hyperlipidemia, diabetes mellitus, and tobacco use.^7^, ^10^, ^62^, ^63^ We investigated the frequency of the abovementioned antemortem vascular risk factors and found no significant differences between TDP‐43(+) and TDP‐43(−) (except for a trend for older age at MRI and death in the former) or between AD‐TDP type‐*α* and type‐*β*. Moreover, we also examined the postmortem cerebrovascular pathologies, including arteriolosclerosis, microinfarcts, large/lacunar infarcts, and CAA (whether analyzed separately or using the vascular composite score) and again found no dissimilarities between TDP‐43(+) and TDP‐43(−) and between AD‐TDP type‐*α* and type‐*β*. This discrepancy between dissimilar WMH volumes between type‐*α* and type‐*β* (with type‐*α* showing almost twice the total WMH volume than type‐*β*) and similar antemortem vascular risk factors and postmortem cerebrovascular pathologies strengthens the validity of our main findings. Of note, prior studies on FTLD‐TDP and WMH also showed that the increase in WMH burden was independent of chronological age, cardiovascular risks, or cerebrovascular pathology.^12^, ^18^, ^57^ Our study hence provides further evidence that AD‐TDP type‐*α* more closely mimics FTLD‐TDP than AD.

In the current study, type‐*β* was not only associated with less WMH burden than type‐*α*, but it also showed less total and subcortical occipital WMH burden, as well as periventricular parietal WMH burden, than TDP‐43(−). Type‐*β* is characterized by the presence of TAT inclusions that share molecular characteristics with NCIs in FTLD‐TDP and AD‐TDP, but with restricted distribution to the limbic regions (with the highest burden found in the amygdala, entorhinal cortex, and hippocampus and least in the non‐limbic occipitotemporal cortex).^25^, ^26^ In our study, less WMH burden in the subcortical occipital lobe could be explained by this typically scarce TAT burden in the occipitotemporal lobe, which potentially leads to less neurodegeneration, axonal loss, and demyelination. Moreover, previous studies on AD ± TDP found that posterior WMHs were more associated with neurodegeneration caused by AD pathology.^14^, ^15^ It is also possible that the association with tau itself acts as either a protective/less destructive mechanism possibly leading to less neurodegeneration, as participants with type‐*β* inclusions were more frequently cognitively intact and older at the time of death.^25^, ^37^ Graff‐Radford et al. ^11^ recently found that WMHs did not associate with tau burden but correlated with greater CAA, resulting in microbleeds. Although type‐*β* typically presents with higher ADNC burden,^37^, ^47^, ^64^ there were no significant differences in ADNC or CAA severity between our type‐*α* and type‐*β* cohort. It is also possible that CAA burden could be lower in type‐*β* compared to TDP‐43(−) potentially explaining the differences in subcortical occipital lobe WMH volumes; however, we did not see this in our cohort (data not shown). Furthermore, periventricular fronto‐parietal WMHs are common in cognitively unimpaired older adults^65^ but can correlate with amyloid‐associated microbleeds and onset of dementia when significantly increased.^11^ It is possible that the less periventricular frontoparietal WMHs seen in type‐*β* could be related to the increased cognitive resilience. Although we did not see this in our current cohort, this can be evaluated in future bigger studies.[Fig alz14516-fig-0005]

The stark differences in the WMH burden of type‐*α* and type‐*β* suggest different pathomechanisms. One possible explanation revolves around the nature of the inclusions themselves. As already mentioned, type‐*α* inclusions are more heterogeneous, with TDP‐43 habitually depositing not only within the neuronal body, but also within its neuronal processes (dendrites and axons) and associated glia, thus representing direct white matter pathology. Contrarily, type‐*β* is characterized chiefly by combined TDP‐43/tau pathology within the neuronal body, with less direct deposition of pathologic TDP‐43 in neurites or glia, which by itself would trigger less white matter damage. Other possible mechanisms leading to increased WMHs in relation to AD‐TDP type‐*α* could be similar to those proposed in FTLD‐TDP, including Wallerian degeneration from TDP‐43 pathology deposition, which leads to axonal loss and demyelination^14^, ^15^, ^16^, ^66^; reactive gliosis and spongiosis causing intra‐myelinic edema^67^; or increased neuroinflammation leading to reactive oxygen species production and oxidative stress. Another possible mechanism is disruption of the BBB, as some type‐*α* inclusions can be seen in perivascular and periependymal regions. Recently, we described a new type of “star‐shaped” TDP‐43 pathology co‐localizing with tau in astrocytes of super‐agers, which are also located in perivascular regions of the amygdala where they likely affect astrocytic foot processes and BBB integrity.^68^

Currently, in vivo biomarkers of AD‐TDP pathology are lacking. Although PET imaging using tracers that bind tau and Aβ are being used in the antemortem diagnosis of AD in both clinical and research settings,^69^, ^70^, ^71^, ^72^, ^73^ there are currently no validated radiotracers able to bind TDP‐43 in vivo, and no biofluid assays for TDP‐43 have been validated. An indirect imaging biomarker available is based on distinct patterns of temporal lobe hypometabolism on FDG‐PET imaging that uses the inferior/medial temporal metabolism ratio to predict hippocampal sclerosis,^74^ and inferior‐to‐medial temporal/frontal supraorbital metabolism ratio to predict AD‐TDP.^75^ Although our study shows evidence of associations between WMHs and AD‐TDP, disentangling the associations between WMHs and other vascular or neurodegenerative diseases hinders any potential utility for WMH quantification/distribution pattern as an independent biomarker for AD‐TDP. However, future studies could investigate its potential utility as an adjunct neuroimaging biomarker that can be used in combination with structural MRI and FDG‐PET signatures to predict postmortem AD‐TDP pathology.

A strength of this study is the large cohort of TDP‐43(−) and TDP‐43(+) and the further characterization of AD‐TDP pathology beyond the simple positive versus negative status. Another important strength is that WMH quantitation was performed automatically with manual correction of segmentation algorithm errors. Limitations include the relatively smaller number of participants with TDP‐43 typing and staging. Indeed, we attempted to assess the relationships between WMH burden and TDP‐43 staging, but these were not included in the study as results yielded only trends, most likely related to poor statistical power. Another limitation is that nearly all our participants were White, which limits the generalizability of our findings.

In summary, different AD‐TDP types have opposite effects on WMH burden. Type‐*α* is associated with a greater WMH burden and closely mimics the effects of FTLD‐TDP rather than AD. Contrarily, type‐*β* is associated with an overall decreased WMH burden. The contrasting effects of type‐*α* and type‐*β* suggest different pathomechanisms.

## CONFLICT OF INTEREST STATEMENT

Dr. Carlos, Mr. Weigand, and Ms. Pham have no disclosures to report. Drs. Petersen, Dickson, Whitwell, and Josephs report receiving research funding from the National Institutes of Health (NIH). Dr. Jack reports receiving research support from NIH, the GHR foundation, and the Alexander Family Alzheimer's Disease Research Professorship of the Mayo Clinic; serving on an independent data monitoring board for Roche; consulting for and serving as a speaker for Eisai; and consulting for Biogen; however, he receives no personal compensation from any commercial entity. Author disclosures are available in the Supporting Information.

## CONSENT STATEMENT

This study was performed following the guidelines from the Declaration of Helsinki and was approved by the Mayo Clinic Institutional Review Board. All participants or their proxies provided written informed consent.

## Supporting information



Supporting information

Supporting information

## Data Availability

Data that support the findings in this study are available from the corresponding author (K.A.J.) upon reasonable request.
