# Supplementary material for: White matter hyperintensities and TDP‐43 pathology in Alzheimer's disease
Source: Alzheimers Dement. 2025 Jan 17;21(2):alz14516. doi: 10.1002/alz.14516 (PMC11851154; doi:10.1002/alz.14516)
Supplement: Supplementary file 1 — Supporting information [file ALZ-21-alz14516-s002.docx]

**SUPPLEMENTARY FILES**

**Supplementary Table 1. Cerebrovascular pathologic findings**

| **Pathology** | **TDP-43(-)** | **TDP-43(+)** | **p-value** | **Type-α** | **Type-β** | **p-value** |
| --- | --- | --- | --- | --- | --- | --- |
| **CAA** |  |  | 0.50 |  |  | 0.69 |
| Absent | 13/73 (18%) | 8/70 (11%) |  | 3/24 (12%) | 0/14 (0%) |  |
| Mild | 24/73 (33%) | 19/70 (27%) |  | 7/24 (29%) | 5/14 (36%) |  |
| Moderate | 25/73 (34%) | 29/70 (41%) |  | 10/24 (42%) | 6/14 (43%) |  |
| Severe | 11/73 (15%) | 14/70 (20%) |  | 4/24 (17%) | 3/14 (21%) |  |
| **Arteriolosclerosis** |  |  | 0.12 |  |  | 0.61 |
| Absent | 8/73 (11%) | 4/71 (6%) |  | 1/24 (4%) | 1/13 (8%) |  |
| Mild | 20/73 (27%) | 11/71 (15%) |  | 2/24 (8%) | 2/13 (15%) |  |
| Moderate | 25/73 (34%) | 36/71 (51%) |  | 15/24 (63%) | 5/13 (38.5%) |  |
| Severe | 20/73 (27%) | 20/71 (28%) |  | 6/24 (25%) | 5/13 (38.5%) |  |
| **Microinfarcts** |  |  | 0.81 |  |  | 0.45 |
| Absent | 54/71 (76%) | 52/70 (74%) |  | 20/25 (80%) | 9/14 (64%) |  |
| Present | 17/71 (24%) | 18/70 (26%) |  | 5/25 (20%) | 5/14 (36%) |  |
| **Lacunar/large infarct** |  |  | 0.50 |  |  | 0.86 |
| Absent | 51/71 (72%) | 48/72 (67%) |  | 16/25 (64%) | 10/15 (67%) |  |
| Present | 20/71 (28%) | 24/72 (33%) |  | 9/25 (36%) | 5/15 (33%) |  |

Data are presented at counts and percentages. P-values are from Chi-squared test or Fisher’s Exact test, as appropriate. Abbreviations: CAA = cerebral amyloid angiopathy; TDP-43 = transactive response DNA-binding protein 43

**Supplementary Table 2. TDP-43 status effect on total and regional WMH**

| Region | Relative WMH volume | 95% CI | | P-value |
| --- | --- | --- | --- | --- |
|  |  | Lower | Upper |  |
| Total | 1.01 | 0.82 | 1.26 | 0.89 |
| PV frontal | 1.00 | 0.81 | 1.23 | 0.98 |
| PV temporal | 0.96 | 0.80 | 1.16 | 0.69 |
| PV parietal | 0.98 | 0.78 | 1.23 | 0.86 |
| PV occipital | 1.05 | 0.91 | 1.23 | 0.49 |
| Deep gray and white | 1.14 | 0.82 | 1.59 | 0.43 |
| SC frontal | 1.40 | 0.90 | 2.17 | 0.13 |
| SC temporal | 0.97 | 0.58 | 1.62 | 0.91 |
| SC parietal | 1.06 | 0.70 | 1.60 | 0.77 |
| SC occipital | 0.86 | 0.70 | 1.05 | 0.14 |

Data are shown as changes in relative WMH burden (with 95 % confidence intervals) associated with TDP-43 positive status. P-values are from penalized linear regression models adjusting for age at MRI and TIV.

Abbreviation: PV = periventricular; MRI = magnetic resonance imaging; SC = subcortical; TIV = total intracranial volume; TDP-43 = transactive response DNA-binding protein 43; WMH = white matter hyperintensity

**Supplementary Table 3: TDP-43 type effect on total and regional WMH**

| Region | Relative WMH volume | 95% CI | | P-value |
| --- | --- | --- | --- | --- |
|  |  | Lower | Upper |  |
| **Type-α relative to TDP-43 negative** | | | | |
| Total | 1.30 | 0.96 | 1.76 | *0.09* |
| PV frontal | 1.23 | 0.91 | 1.67 | 0.17 |
| PV temporal | 1.11 | 0.86 | 1.44 | 0.42 |
| PV parietal | 1.16 | 0.84 | 1.59 | 0.36 |
| PV occipital | 1.11 | 0.90 | 1.38 | 0.33 |
| Deep gray and white | 1.75 | 1.11 | 2.77 | **0.02** |
| SC frontal | 2.19 | 1.15 | 4.17 | **0.02** |
| SC temporal | 1.94 | 0.98 | 3.83 | *0.06* |
| SC parietal | 1.59 | 0.89 | 2.83 | 0.11 |
| SC occipital | 1.00 | 0.76 | 1.33 | 0.99 |
| **Type-β relative to TDP-43 negative** | | | | |
| Total | 0.70 | 0.49 | 1.01 | *0.06* |
| PV frontal | 0.74 | 0.52 | 1.07 | 0.11 |
| PV temporal | 0.79 | 0.58 | 1.08 | 0.13 |
| PV parietal | 0.72 | 0.49 | 1.06 | *0.09* |
| PV occipital | 0.97 | 0.75 | 1.26 | 0.83 |
| Deep gray and white | 0.68 | 0.39 | 1.18 | 0.17 |
| SC frontal | 0.68 | 0.31 | 1.48 | 0.32 |
| SC temporal | 0.61 | 0.27 | 1.40 | 0.24 |
| SC parietal | 0.66 | 0.33 | 1.34 | 0.25 |
| SC occipital | 0.69 | 0.49 | 0.96 | **0.03** |
| **Type-α relative to type-β** | | | | |
| Total | 1.79 | 1.18 | 2.70 | **0.01** |
| PV frontal | 1.60 | 1.06 | 2.42 | **0.02** |
| PV temporal | 1.38 | 0.97 | 1.96 | *0.07* |
| PV parietal | 1.56 | 1.01 | 2.40 | **0.04** |
| PV occipital | 1.14 | 0.85 | 1.53 | 0.39 |
| Deep gray and white | 2.44 | 1.31 | 4.55 | **0.01** |
| SC frontal | 3.03 | 1.26 | 7.28 | **0.01** |
| SC temporal | 2.94 | 1.17 | 7.43 | **0.02** |
| SC parietal | 2.27 | 1.04 | 4.96 | **0.04** |
| SC occipital | 1.41 | 0.97 | 2.07 | *0.07* |

Data are shown as changes in relative WMH burden (with 95 % confidence intervals) associated with TDP-43 positive status. P-values are from penalized linear regression models adjusting for age at MRI and TIV. P-values that are significant (<0.05) are shown in bold; those that represent a trend (<0.1) are shown in italics.

Abbreviation: PV = periventricular; MRI = magnetic resonance imaging; SC = subcortical; TIV = total intracranial volume; TDP-43 = transactive response DNA-binding protein 43; WMH = white matter hyperintensity
